# Social Isolation of Horses vs. Support Provided by a Human

**DOI:** 10.3390/ani15111649

**Published:** 2025-06-03

**Authors:** Iwona Janczarek, Izabela Gazda, Joanna Barłowska, Julia Kurnik, Jarosław Łuszczyński

**Affiliations:** 1 Department of Horse Breeding and Use, Faculty of Animal Sciences and Bioeconomy, University of Life Sciences in Lublin, Akademicka 13 Str., 20-950 Lublin, Poland; iwona.janczarek@up.lublin.pl (I.J.); izabelagazda304@gmail.com (I.G.); juliakurnik@o2.pl (J.K.); 2 Department Quality Assessment and Processing of Animal Products, Faculty of Animal Sciences and Bioeconomy, University of Life Sciences in Lublin, Akademicka 13 Str., 20-950 Lublin, Poland; joanna.barlowska@up.lublin.pl; 3 Department of Genetics, Animal Breeding and Ethology, Faculty of Animal Science, University of Agriculture in Cracow, Al. Mickiewicza 21 Str., 31-120 Cracow, Poland

**Keywords:** horse, social isolation, social support, HRV, behaviour

## Abstract

It is assumed that horses have a strong herd instinct that cannot be suppressed by any form of social support from humans. The aim of this study was to assess the behaviour and heart rate parameters of horses that received various forms of human support during short-term social isolation. The research material consisted of 12 clinically healthy recreational warmblood mares and geldings. The test was carried out four times. The research test consisted of two parts, which began in the presence of accompanying horses and under conditions of social isolation, with and without support provided by a human (types of contact: tactile, vocal, tactile and vocal). Behavioural observations were carried out on a five-point scale, and the heart rate parameters were measured using Polar ELECTRO OY Kempele (Finland) measuring devices (Polar H9 type) and Polar Ignite2 receivers. Data analysis was carried out using the Polar Flowsync application coupled with the Polar Flow service. It was concluded that social support provided by humans in tactile, vocal, or tactile and vocal forms during the isolation of horses from the herd does not have very significant effects, especially in mares. The geldings respond much more favourably than the mares. In addition, at this point in the research, it can be suggested that vocal support may have the best effects. The more emotionally balanced the horses are, the more pronounced these effects should be.

## Simple Summary

Horses’ behaviour and heart rate during short-term isolation with and without social support provided by a human was studied. During the test with no support, the human only held the horse on a rope. With support, the human stroked the horse. In the next repetition of the test, the human spoke to the horse, and after that, he stroked and spoke at the same time. The horses’ behaviour was observed at this time, and their heart rates were measured. It was found that no type of social support provided by humans had sufficiently desired effects, especially in mares. However, speaking to a horse was better than other types of support or the lack of support, but mainly in calm horses.

## 1. Introduction

Equines are a species characterised by a well-developed herd instinct and robust social connections [1,2,3]. Living in a herd enhances the perception of security, facilitates defence mechanisms, and promotes the formation of social and emotional bonds [4,5,6]. The domestication process of horses is continued by the employment of specific forms of their use and requires humans to attend to the vital needs of horses to ensure their optimal health and overall satisfaction [7,8,9]. All forms of social isolation, inter alia, disturb the nature of horses and lead to problems in their cooperation with humans [10]. What seems particularly dangerous is occasional displays of aggression [11,12] or, on the contrary, apathy and the associated unwillingness to work [13].

Isolation can arise from various factors in the lives of modern horses. These include housing and paddocking systems, usage methods, personality traits, age, and sex of the animal. Additionally, factors such as transport, participation in competitions, illness, convalescence, and mental disorders can contribute to this issue. Unfortunately, isolation has become an inseparable aspect of their lives [2,13,14,15,16,17,18,19,20,21]. It is crucial to emphasise that the most harmful form of social isolation is the inability to make eye contact or, even more significantly, to establish auditory contact [22,23]. Many horses are still kept in solitude, often without the companionship of other animals, relying solely on humans for social interaction [24]. As a result of these circumstances, horses must adapt to a new way of life for their survival, a process referred to as allostasis [25]. An inherent desire to live, combined with an inability to make necessary changes, does not lead to satisfaction [26,27].

The negative emotions that accompany social isolation may lead to apathy, anxiety, and aggression, as mentioned earlier. One of the consequences is the gradual build-up of stress, which causes hormonal disturbances in the body [19,28]. This phenomenon is primarily associated with the excessive secretion of cortisol from the adrenal cortex. Initially, it mobilises the body’s resources, but over time, it gradually leads to its destruction. This can result in somatic diseases, emotional disorders such as unhealthy habits or stereotypy, and, in severe cases, even death [29]. These unpleasant consequences of isolation have prompted a search for various solutions to mitigate its effects [20,30,31,32,33]. The methods applied nowadays still include the use of mirrors, in which the horse can see its own reflection, as well as the use of toys, slow feeders, and music therapy [21,30,32,34,35].

The topic of social support in interactions among individuals of the same or different species is attracting increasing interest from researchers [2,36,37]. The term “social” generally refers to fundamental life issues important to every individual [38]. It is also commonly associated with the idea of providing support [39]. Social support is a specific type of social interaction that primarily involves people [40]. It also refers to the various forms of assistance, understanding, and solidarity that individuals provide to one another in difficult life situations. The purpose of social support is to improve quality of life and strengthen social connections. It plays a crucial role in developing positive emotions and overall well-being [41]. Some individuals gain more from social support from other species than from their own kind, while animals predominantly provide support within their own species [29,37,42]. For example, the basic role of social support for horses is served by the herd or other individuals [43]. Horses in the company of goats still cope poorly with social isolation [36], and the company of domestic cattle provides them with no support at all [44].

It is worth noting that the character and relationships between animals can vary depending on the individual characteristics of each animal. Some horses may prefer a quiet environment and avoid other animals, whereas others may benefit from interacting with different species. It is important to monitor these interactions and make sure that all animals feel comfortable and safe in their environment. Relationships with humans have a particular impact on the development of positive emotions in horses [43,45]. Horses, being social animals, are sensitive to interactions with humans, whom they should regard as superior but friendly members of their own herd, thanks to the process of domestication and raising [46,47,48,49,50,51,52]. Positive and stable relationships with people are often the key to experiencing various aspects of well-being, including a sense of security, trust and understanding, which fosters positive emotions [53]. Caresses, gentle touches, kind words, and rewards during training are important for building positive emotions. Positive relationships between humans and horses, characterised by calmness, patience and empathy, can reduce stress and anxiety [54] while enhancing the effectiveness of training these animals. Unusual situations, such as transport, therapy, health problems or specific uses, necessitate social support for horses from humans [13,14,15,16,17,18,19,20,21,47,55]. A response to isolation from fellow animals is one of the most challenging aspects of these unusual situations, and support from other animal species and humans may not always be sufficient.

It was assumed that horses have a strong herd instinct that cannot be suppressed by any form of social support provided by humans. With this assumption in mind, the aim of this study was to assess the behaviour and heart rate parameters of horses during short-term social isolations while receiving various types of human support.

## 2. Materials and Methods

### 2.1. Approval from the Local Animal Research Ethics Committee

Pursuant to the Act of 15 January 2015 on the protection of animals used for scientific or educational purposes, research requires approval if it “involves pain, suffering, distress or permanent damage to an animal equal to or worse than a needle prick”. The observations described do not exhibit the above-cited characteristics and were conducted with the consent of the animal owners as routine measures desensitising horses to environmental stimuli.

### 2.2. Horses

The research material consisted of 12 clinically healthy Malopolski horses, including five mares and seven geldings. The average horse age was 9.70 ± 1.36 years. The horses had been kept in one building for at least 24 months, in stalls with 2.2 m high solid partition walls. Each stall with dimensions of 3 × 3 m was equipped with a corner plastic feeding trough, an automatic metal watering trough, and a salt lick on a plastic stand. The floor in the stall was bedded with straw on a daily basis. The horses had constant access to hay suspended in special nets near the watering troughs. In addition, they were fed complete concentrated feed three times a day, according to individual indications. The horses were handled on a daily basis (feeding, cleaning, and walking them to and from the paddocks) by two caretakers employed for a minimum of 12 months. The horses were permanently used for recreational purposes for six days a week, for 2–3 h a day. Each horse was mounted and handled before/after the ride by 8–10 different people (persons unfamiliar/familiar to the horses, children/adults, females/males, and persons with different levels of equestrian ability). For the rest of the time and on the days off, the horses stayed in stalls, in paddocks or on pastures. The animals spent at least four hours a day in the paddock or on a pasture. The horses had not been previously socially isolated.

Based on an interview with the caretakers, behavioural disorders were initially excluded in the horses. On the day before the start of the study, the horses were subjected to a primary medical examination and found to be clinically healthy.

### 2.3. The Course of the Social Support Test

The test was carried out four times in the afternoon on four successive days off for the horses, i.e., every week (stages 1, 2, 3, and 4). In each stage, the caretaker led the test horses one by one to a designated place near the stable while keeping them on a 2 m long rope. The test, which comprised two parts (A and B), was then started. The beginning of part A of the test involved the presence of accompanying horses: at the same time, two horses that did not participate in the test (accompanying horses) were led up to the test horse. Each time, these were the same horses. The horses (a mare and a gelding) were familiar with the test horses and had never been in conflict with them. During the test, they were located 1–2 m away from the test horse. Although the horses were held on a rope by their caretakers, they were not allowed to maintain tactile contact with the test horse. After three minutes of the test, the accompanying horses were led back to the stable. Then, part B of the test was started, which involved social isolation without or with tactile/vocal support provided by a human. Each time, the test horse was kept at the same place as in part A of the test for another three minutes. In the first stage, the caretaker had no tactile or vocal contact with the horse (variant 1: control test). In the second stage, the caretaker’s task involved stroking the horse around the neck and shoulder, but still without vocal contact (variant 2: tactile contact). In the third stage, the caretaker only maintained vocal contact, speaking to the animal in a calming voice (variant 3: vocal contact). In the fourth stage, the caretaker maintained both vocal and tactile contact with the horse (variant 4: vocal and tactile contact). The order of selection of the test variant for each horse was determined using the Cartesian Square method. The method eliminated the possible impact of the horses being accustomed to the course of the test on the results obtained.

### 2.4. Research Methods

On each of the four research days (four successive days off for the horses), the horses were examined at rest (in their own stalls) and in parts A and B (one of the four variants) of the test. For each horse, examinations of the heart rate parameters and behavioural observations were then carried out. The examination and observations at rest, in parts A and B of the test, lasted for 3 min.

Behavioural assessments were carried out based on the authors’ original ethogram prepared for the purposes of the study (Table 1).

The heart rate parameters were measured using Polar ELECTRO OY (Kempele, Finland) measuring instruments. The horses had previously been accustomed to these instruments. A POLAR TF H10+ Crush heart rate monitor compatible with smartphones via Bluetooth was used. Data analysis was carried out using the Polar Beat 3.5.6 application for Android, coupled with the Polar Flow service. The following were evaluated: heart rate (HR) per minute; rMSSD: square root of the average sum of the squares of the differences between successive RR intervals [ms], indicating the activity of the parasympathetic part of the autonomic nervous system (ANS); LF: a low-frequency spectrum in the range of 0.04–0.15 Hz (low frequency), dependent on changes in ANS fibres (ms^2^); and HF: a high-frequency spectrum in the range of 0.16–0.4 Hz (high frequency), which is responsible for the influence of breathing on the heart rate, and depends on the modulation of the parasympathetic part of the ANS (ms^2^).

### 2.5. Statistical Methods

The statistical analysis used Statistica for Windows 13.0 software (TIBCO Software, Inc., Palo Alto, CA, USA). The normal distribution of the variables was assessed using the Shapiro–Wilk and Lilliefors tests. After checking the homogeneity of variance (Levene’s test), the variables for which compliance with the normal distribution was noted (Table 2) were analysed using single-factor variance analyses. The significance of differences between the mean values was determined using Tukey’s (RIR) test. The variables for which no compliance with the normal distribution was identified were analysed using non-parametric tests. For the comparison of dependent samples (repeated sampling) in stages (1, 2, 3, 4) or at rest and in parts A and B of the test, Friedman’s rank test was applied. For the comparison of independent samples, the Mann–Whitney U test was used for the sex (mare, gelding). In all cases, a significance level of *p* ≤ 0.05 was adopted.

## 3. Results

### 3.1. Heart Rate Parameters

**Figure 1:**
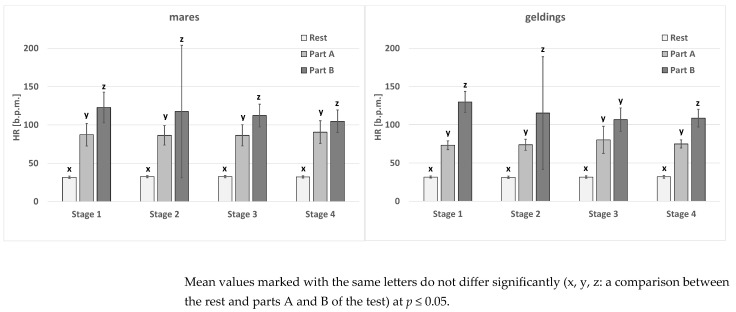
The HR parameter in the test horses.

In the case of the geldings at rest and the mares and the geldings in part A of the test, the HR parameter did not differ significantly between the stages (Table 3). In mares, the resting values differed between stages 1 and 2. However, in stages 3 and 4, the values were similar to those in stages 1 and 2. In the mare group, in part B, no significant differences were noted. In the gelding group, in part B, significant differences were noted between stage 1 and stages 2–4. Between the rest and the subsequent parts of the test, significant differences occurred in each case, with the exception of the geldings in the third stage of the test (no differences between parts A and B) (Figure 1). Sex-related differences were noted in stages 1 and 4 in part A of the test (Table 3).

In the mare group at rest and in part B of the test, no significant differences were noted for the mean values of the rMSSD parameter between the stages (Table 4). Significant differences in part A of the test also did not occur for geldings. In this sex group, the differences at rest occurred between stages 1, 3, 4, and 2. In addition, the results in stage 2 did not differ from those in stage 3. Part B of the test for the mares and geldings observed no significant differences between the mean values.

**Figure 2:**
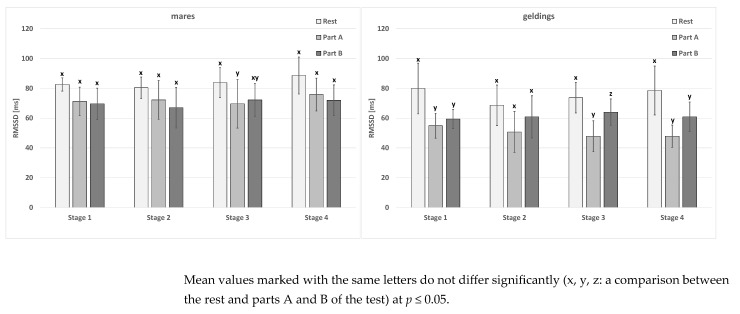
The RMSSD parameter in the test horses.

The differences between the rest and parts A and B of the test occurred primarily in the third stage of gelding testing (Figure 2). Moreover, they were noted between the two parts of the test and the rest in the first and fourth stages in the gelding group. For mares, differences also occurred between the rest and part A of the test in stage 3. In contrast, sex-related differences occurred in each stage in part A of the test (Table 4).

In the mare and gelding group at rest, no significant differences occurred between the stages in the mean values of the LF parameter (Table 5). As for the mares in part A of the test, no significant differences between the mean values were noted. In the gelding group, significant differences were recorded between stages 1 and 3. Part B of the test for the mares and geldings revealed no significant differences between the mean values.

**Figure 3:**
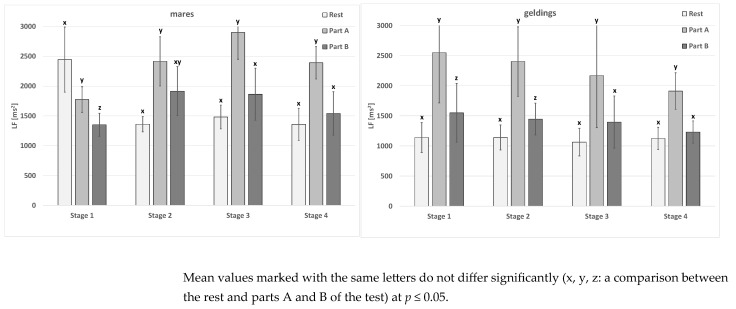
The LF parameter in the test horses.

Significant differences between the rest, part A, and part B of the test were noted in all cases, but primarily in geldings in stages 1 and 2 and in mares in stage 1 (Figure 3). However, the differences between the two mean values, similar to each other, at rest and in part B of the test and part A concerned all the other cases, with the exception of the mares in stage 2. The sex-related differences concerned the rest in stage 3, part A of the test in stage 4, and part B of the test in stage 2 (Table 5).

No significant differences between the stages in terms of resting HF were noted in the mare group (Table 6). The value of this parameter in geldings in stage 4 was, however, significantly lower than the values from the other stages. In the mare group, in part A of the test, no significant differences occurred. In the gelding group, the mean value of this parameter in stage 1 differed significantly from the values from stages 3 and 4. In addition, the value from stage 2 was similar to that from the other stages. No differences were noted between the stages in part B of the test of mares and geldings.

**Figure 4:**
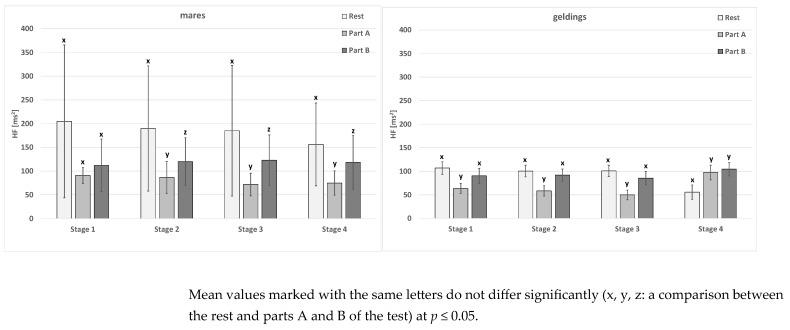
The HF parameter in the test horses.

Differences between the rest, part A, and part B of the test were not only noted in the mares in stage 1 (Figure 4). In addition, the mean value of the parameter in the two sex groups in part A of the test was most frequently significantly lower than the resting values, similar to each other, and the values from part B of the test. An exception was the geldings tested in stage 4. No sex-related differences occurred (Table 6).

### 3.2. Rating for Behaviour

**Figure 5:**
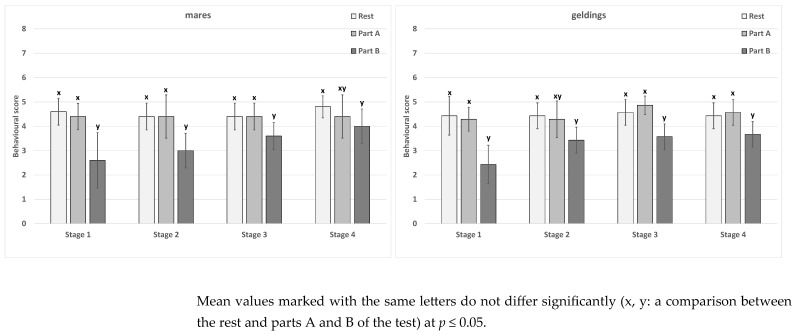
The score for the behaviour of the test horses.

The score for behaviour in the subsequent stages did not differ significantly in the two sex groups (Table 7). In the mare group, significant differences were also not noted in part A of the test. In contrast, as for the geldings, differences in part A occurred between stages 1 and 2 and stage 3. In stage 4, the results were similar to those of the other stages. In part B, in the mare group, differences were noted between stage 1 and stage 4. The differences in the gelding group occurred between stage 1 and stages 2–4. Differences between the rest and parts A and B of the test were noted for each stage, both in the mares and the geldings (Figure 5). No sex-related differences were recorded (Table 7).

## 4. Discussion

The discussion of the results begins with the HR, which, when not considering the exertion component, is a good indicator of an emotional response [56,57,58]. It appears that the resting HR value alone indicated a lower emotional stability in mares, compared to geldings. These results are not surprising, as they are consistent with those published by Janczarek et al. [59], Janczarek et al. [60], and Saslow [61]. These authors indicated that the emotional stability of mares is labile, depending, among other things, on the sexual cycle or general hormonal balance. For this reason alone, mares are not willingly used in equestrian sports [62,63,64].

However, the situation was slightly different for the resting RMSSD value. This parameter indicates the activity of the parasympathetic part of the autonomic nervous system, thus determining the degree of relaxation and even satisfaction of the body [36,60]. Its high values are therefore desirable for health and well-being. The results of the current study show that the mares indeed did not differ in this regard from the geldings, yet the values noted in them also did not differ between the stages. In the geldings, this parameter was significantly lower for two out of four stages than in the other stages. It is difficult to interpret these results unambiguously. It can be assumed, however, that the above-mentioned labile emotional excitability does not necessarily go hand-in-hand with the overall relaxation of the body. It is likely that HR correlates even with a very discreet impact on the environment without changing the overall emotional state. These results are, therefore, consistent with those published by Lenoir et al. [65] and Rietmann et al. [66]. These authors emphasised that assessing emotions using HR as the sole heart rate parameter may be imprecise.

Similar results were obtained during the HF analysis, but with one exception that appeared in the fourth stage of gelding testing. At that time, the parameter decreased significantly in relation to the three other stages. This was a surprising value since it did not coincide with the results of any of the other parameters. The HF, similarly to the RMSSD, indicates the parasympathetic activity of the ANS [36,60]. This activity, however, was examined using another method, which may be the reason for obtaining different results. A similar method was employed to measure the sympathetic–parasympathetic activity of the ANS [67,68,69]. Its determinant was the LF parameter, and although the results did not differ for it between the stages, one case of sex-related differences was noted. In the third repetition of the test, the mares had a higher score than the geldings, which again, as in the case of the HR, may indicate their greater emotional excitability.

Although the current results show that leading the horse out of the stable and having visual contact with other horses caused an increase in HR irrespective of sex, this further indicated that the mares’ emotional excitability is greater than that of the geldings. Since the constant changeability of the living environment is an indispensable part of the life of most modern horses, the response to this fact appears to be very important, not only for maintaining the welfare of these animals but also for their use by humans.

Another important issue is the isolation and the social support provided by humans. Social isolation affects horses as well, although it is most often a stressful experience, particularly for young or unaccustomed animals [2,19,70,71,72]. In the current study, isolation resulted in a significant increase in HR, which was a predictable result. Although the mares and the geldings responded in a similar way, it is worth analysing what happened later on. The results show that social support provided by a human only worked for the geldings, but not in every stage of the test. When the human maintained vocal contact with the horse, or when this contact was vocal and tactile at the same time, the geldings were calmer than in isolation with no support provided by a human or with support involving tactile contact. Similar results were also noted for the RMSSD. However, the mares did not respond significantly to the support.

In today’s world, in which a human can occasionally be the sole companion of a horse, this situation raises concern. It can, however, be explained in two ways. Firstly, it indicates that mares are more independent than geldings, which is reflected in, e.g., greater nervousness, aggressiveness, or reactivity, as indicated by the study conducted by Fenner et al. [63]. Secondly, such a response may be associated with herd hierarchy, which, in the natural environment, is actually only formed by mares. Perhaps, due to this fact, a human can become more of a companion to a gelding than to a mare. These results are at least partly consistent with the information published by Aune et al. [62].

Slightly different results were obtained for the HF, where the mares also responded to the support during isolation. In fact, in the case of the geldings, the parameter was noted to return to the resting level. Surprisingly, this also occurred when no support was provided by a human. Tactile or vocal support also appeared to be favourable. However, the simultaneous use of tactile support and vocal support appeared to be unfavourable. It is difficult to interpret these results. Should the support provided by a human be discreet, or is this discrepancy due to the measuring method employed? In addition, in the case of the geldings, each type of support caused an increase in the parameter concerned in relation to the part of the test where the horses were accompanied by other horses. It is difficult to explain the discrepancy between the results for the RMSSD and HF.

This study examined the fluctuations in LF values during isolation. Notably, this parameter showed a decrease, reaching values comparable to the resting levels during the provision of each type of human support. Furthermore, these values rarely exceeded the resting levels and were never higher than those observed when the test horse was accompanied by other horses. It can be assumed that contact between horses sometimes triggers much stronger emotions than even short-term isolation from the herd but in the company of a human. This study confirms that humans may, at least partially, be considered companions for modern horses, as also indicated by studies conducted by Koski and Spännäri [73] and by Kelly et al. [47].

Linking heart rate results to behaviour ratings proves challenging, particularly since the scores during isolation were significantly lower compared to others, regardless of whether support was provided. While a distinct positive effect of support—especially vocal or vocal and tactile contact—was noted, it lacked the strength to impact behaviour observed at rest or during interactions with other horses. It is difficult to interpret these results unambiguously, as a simultaneous examination of HRV and behaviour yields, in general, different results [74]. These authors point to the masking of actual emotions by the horses and the demonstration of desirable behaviour, which is due to the process of being raised by humans. The current study did not confirm this fact for the mares or geldings. Apparently, the tested group of horses was not ideally raised or was characterised by considerable emotional excitability, which made them behave more poorly than indicated by the HRV parameters. This fact is mainly confirmed by the resting results for behaviour and the occurring differences between certain HRV parameters, which were lower than those obtained during a similar study conducted by Janczarek et al. [60].

In summary of the discussion of our results, we emphasise that this study included only 12 Małopolska horses, with 5 mares and 7 geldings in the sample. We did not study stallions or young horses, nor the influence of different environments or human involvement in the upbringing and training of the horses. Therefore, we do not know what results would have been obtained if the factors or their levels that we did not investigate had been included. We consider this to be a limitation of our study.

## 5. Conclusions

Social support provided by a human in the tactile, vocal, or tactile and vocal form during the isolation of horses from the herd does not present very notable effects, especially in mares, although the geldings responded much more favourably than the mares. Furthermore, at this stage of the research, it can be proposed that vocal support may yield the most significant effects. The more emotionally balanced the horses are, the more pronounced these effects are likely to be. Due to the limited number of adult horses of only one breed, the lack of consideration of stallions, and the influence of different environments, further research in this area should be continued.

## Figures and Tables

**Table 1:** The method of assessing the horses’ behaviour at rest and during the social support test.

Score	Characteristic Behaviour of the Horse
1	Horse moves away from the caretaker to the distance of the rope length, anxiety reaction of over 15 s. Horse jumps back and sideways several times, exhibits considerable anxiety (ears laid back, head tossing, head raised, tail swishing, defecation, vocalisation). Caretaker has difficulty holding the animal.
2	Horse moves away from the caretaker to the distance of the rope length, anxiety reaction ranging from 11 to 15 s. Horse jumps back or sideways once, exhibits considerable anxiety (ears laid back, head tossing, head raised, tail swishing).
3	Horse moves away from the caretaker for a distance longer than half the rope length but shorter than the rope length, anxiety reaction ranging from 6 to 10 s. Horse requires strong lead, moves back, accelerates the pace, walks sideways (low body tension, medium head carriage, less lip movement).
4	Horse moves away from the caretaker for a distance no longer than half the rope length, anxiety reaction of no more than 5 s, but the caretaker does not experience difficulties in holding the animal (low body tension, neck height below withers, ears pricked, neutral tail posture).
5	Horse does not move away from the caretaker, no anxiety reaction from the horse (no body tension, neck height below withers, ears pricked, neutral tail posture).

**Table 2:** The variables for which compliance with the normal distribution was noted.

Sex	Factor	Variables
Mares	Stage	HR, RMSSD, LF, HF in parts A and B, and for LF and HF at rest
	Part	HR in stage 3, RMSSD in stages 1, 2, and 4, LF in stages 2, 3, and 4
Geldings	Stage	HR in parts A and C, RMSSD in parts A and B, LF at rest
	Part	RMSSD in stages 2 and 4, LF in stage 4, HF in stage 3
Mares/geldings	Sex	HR, RMSSD, LF in part A in stage 1, HR, RMSSD, LF, HF in part A in stage 2, RMSSD, LF in part A in stage 3, HR, RMSSD, LF in part A in stage 4
		HR, RMSSD, LF, HF in part B in stage 1, HR, RMSSD, LF in part B in stage 2, HR, RMSSD, LF, HF in part B in stage 3, HR, RMSSD, LF, HF in part B in stage 4
		RMSSD, LF at rest in stage 1, RMSSD, LF, HF at rest in stage 2, HR, LF, HF at rest in stage 3, HR, RMSSD, LF, HF at rest in stage 4

**Table 3:** The HR parameter in the test horses.

Stage	Rest		Part A		Part B	
	Mean	SD	Mean	SD	Mean	SD
1.	Mares					
	31.60 ^a^	1.52	87.20 ^a^*	14.79	122.80 ^a^	19.94
	Geldings					
	31.57 ^a^	1.39	73.14 ^a^*	5.70	129.71 ^a^	13.77
2.	Mares					
	32.40 ^b^	1.34	86.40 ^a^	12.70	117.40 ^a^	86.40
	Geldings					
	31.29 ^a^	1.49	73.71 ^a^	7.25	115.29 ^b^	73.71
3.	Mares					
	32.40 ^ab^	1.52	86.40 ^a^	13.80	112.20 ^a^	14.75
	Geldings					
	31.57 ^a^	1.51	80.14 ^a^	17.73	106.57 ^b^	15.31
4.	Mares					
	32.00 ^ab^	1.41	90.60 ^a^*	14.84	104.80 ^a^	14.75
	Geldings					
	31.86 ^a^	1.77	74.86 ^a^*	5.30	108.57 ^b^	11.57

Mean values marked with the same letters do not differ significantly (a, b: a comparison between the stages) at *p* ≤ 0.05. Mean values marked with the same symbols differ significantly (*: a comparison between the sexes in the stages) at *p* ≤ 0.05.

**Table 4:** The RMSSD parameter in the test horses.

Stage	Rest		Part A		Part B	
	Mean	SD	Mean	SD	Mean	SD
1.	Mares					
	82.60 ^a^	4.45	71.20 ^a^*	9.52	69.60 ^a^	10.45
	Geldings					
	79.86 ^a^	16.98	54.86 ^a^*	8.37	59.43 ^a^	6.45
2.	Mares					
	80.40 ^a^	7.16	72.20 ^a^*	13.03	67.00 ^a^	13.64
	Geldings					
	68.57 ^b^	13.59	50.71 ^a^*	13.78	60.86 ^a^	14.17
3.	Mares					
	83.80 ^a^	10.06	69.60 ^a^*	16.32	72.20 ^a^	10.96
	Geldings					
	73.71 ^ab^	10.23	47.86 ^a^*	10.40	64.00 ^a^	8.92
4.	Mares					
	88.60 ^a^	12.40	75.80 ^a^*	10.96	72.00 ^a^	10.32
	Geldings					
	78.57 ^a^	16.39	47.86 ^a^*	7.47	60.86 ^a^	9.96

Mean values marked with the same letters do not differ significantly (a, b: a comparison between the stages) at *p* ≤ 0.05. Mean values marked with the same symbols differ significantly (*: a comparison between the sexes in the stages) at *p* ≤ 0.05.

**Table 5:** The LF parameter in the test horses.

Stage	Rest		Part A		Part B	
	Mean	SD	Mean	SD	Mean	SD
1.	Mares					
	2444.80 ^a^	546.45	1775.40 ^a^	214.69	1351.60 ^a^	193.21
	Geldings					
	1140.143 ^a^	247.69	2550.00 ^a^	834.25	1550.86 ^a^	488.31
2.	Mares					
	1360.60 ^a^	127.62	2416.00 ^a^	412.99	1916.40 ^a^*	410.90
	Geldings					
	1141.29 ^a^	207.38	2402.43 ^ab^	576.85	1447.14 ^a^*	264.59
3.	Mares					
	1481.40 ^a^*	199.53	2900.00 ^a^	449.26	1864.20 ^a^	437.39
	Geldings					
	1064.14 ^a^*	229.07	2168.71 ^b^	864.80	1397.4 ^a^	433.45
4.	Mares					
	1359.00 ^a^	268.84	2391.60 ^a^*	271.26	1542.00 ^a^	363.51
	Geldings					
	1123.57 ^a^	185.68	1912.86 ^ab^*	303.72	1231.43 ^a^	185.44

Mean values marked with the same letters do not differ significantly (a, b: a comparison between the stages) at *p* ≤ 0.05. Mean values marked with the same symbols differ significantly (*: a comparison between the sexes in the stages) at *p* ≤ 0.05.

**Table 6:** The HF parameter in the test horses.

Stage	Rest		Part A		Part B	
	Mean	SD	Mean	SD	Mean	SD
1.	Mares					
	204.80 ^a^	161.12	90.60 ^a^	16.62	112.00 ^a^	55.20
	Geldings					
	106.86 ^a^	13.32	63.51 ^a^	10.74	90.14 ^a^	16.02
2.	Mares					
	189.80 ^a^	131.91	86.60 ^a^	33.59	119.80 ^a^	50.18
	Geldings					
	100.43 ^a^	12.08	58.43 ^ab^	10.95	91.86 ^a^	12.55
3.	Mares					
	185.00 ^a^	137.62	71.80 ^a^	23.78	122.80 ^a^	53.15
	Geldings					
	100.71 ^a^	11.93	49.86 ^b^	10.35	85.43 ^a^	14.10
4.	Mares					
	156.20 ^a^	87.28	74.80 ^a^	25.46	118.20 ^a^	56.53
	Geldings					
	55.71 ^b^	15.22	97.29 ^b^	15.42	104.43 ^a^	14.19

Mean values marked with the same letters do not differ significantly (a, b: a comparison between the stages) at *p* ≤ 0.05.

**Table 7:** The score for the behaviour of the test horses.

Stage	Rest		Part A		Part B	
	Mean	SD	Mean	SD	Mean	SD
1.	Mares					
	4.60 ^a^	0.55	4.40 ^a^	0.54	2.60 ^a^	1.14
	Geldings					
	4.43 ^a^	0.79	4.29 ^a^	0.49	2.43 ^a^	0.79
2.	Mares					
	4.40 ^a^	0.55	4.40 ^a^	0.89	3.00 ^ab^	0.71
	Geldings					
	4.43 ^a^	0.53	4.29 ^a^	0.75	3.43 ^b^	0.53
3.	Mares					
	4.40 ^a^	0.55	4.40 ^a^	0.55	3.60 ^ab^	0.55
	Geldings					
	4.57 ^a^	0.53	4.86 ^b^	0.38	3.57 ^b^	0.53
4.	Mares					
	4.80 ^a^	0.45	4.40 ^a^	0.89	4.00 ^b^	0.71
	Geldings					
	4.43 ^a^	0.53	4.57 ^ab^	0.53	3.67 ^b^	0.52

Mean values marked with the same letters do not differ significantly (a, b: a comparison between the stages) at *p* ≤ 0.05.

## Data Availability

The data presented in this study are available on request from the corresponding author.
